# Estimating the effectiveness of electric vehicles braking when determining the circumstances of a traffic accident

**DOI:** 10.1038/s41598-023-47123-7

**Published:** 2023-11-14

**Authors:** Andrii Kashkanov, Andriy Semenov, Anastasiia Kashkanova, Natalia Kryvinska, Oleg Palchevskyi, Serhii Baraban

**Affiliations:** 1 Faculty of Machine Building and Transport, Vinnytsia National Technical University, Vinnytsya, 21021 Ukraine; 2 Faculty of Information Electronic Systems, Vinnytsia National Technical University, Vinnytsya, 21021 Ukraine; 3 Department of Information Systems, Faculty of Management, Comenius University in Bratislava, Bratislava, 82005 Slovakia; 4 Faculty of Computing and Telecommunications, Poznan University of Technology, 60965 Poznan, Poland; 5 Faculty of Intelligent Information Technology and Automation, Vinnytsia National Technical University, Vinnytsya, 21021 Ukraine

**Keywords:** Mechanical engineering, Applied mathematics

## Abstract

In the vast majority of cases, the braking process is used to prevent traffic accidents. The effectiveness of this process depends on the design and functionality of vehicle braking systems (presence of anti-lock braking system, emergency braking system, preventive safety systems, etc.) and is limited by the amount of frictional forces in contact of tires with the road. The improvement of methodical approaches to evaluating the effectiveness of braking of cars contributes to increasing the accuracy and objectivity of establishing the circumstances of the occurrence of emergency situations. The paper analyses existing methods of evaluating the braking parameters of vehicles (including those with an electric drive) and modern methods of evaluating electric vehicle braking parameters and conducting auto-technical investigations of traffic accidents, which relate to using different methodological approaches and digital technologies at all stages of expert research. In contrast to existing models, the proposed mathematical model for estimating the trajectory of two-axle cars during braking allows for considering various types of input parameter uncertainty, reducing the range of possible modeling errors by 39%. Comparing simulation results and experimental data showed that the average relative error is 4.58%, and the maximum error did not exceed 7.82%. The performed study of the stability of the electric vehicles' movement during emergency braking with the help of developed mathematical models in the Mathcad software environment reveals the content of the algorithm of a similar calculation in specialized computer programs of auto technical examination. Conducting such calculations is relevant in the analysis of real accident situations, where specific circumstances and features that cannot be considered during modeling in specialized software must be taken into account. Simultaneously, the probability of type I errors is reduced by 2–19%, and type II errors are reduced by 43–68%.

## Introduction

The study of aspects related to transportation processes and traffic accidents is grounded in the analysis of the intricate interplay within the 'driver-car-road-environment' (DCRE) system. A road accident can be defined as a disruption in the DCRE system's interaction^1,2^. During the analysis of road accidents, each incident undergoes a comprehensive scientific and technical examination separately^3,4^. Each emergency situation has distinct characteristics, necessitating the determination of partial cause-and-effect, functional, technical, temporal, and other relationships through engineering analysis^5,6^. The main difficulties in solving this kind of problem include:

1. To justify an objective decision about the causes of an accident, many factors should be considered. Most emergency situations are characterized by the simultaneous action of several types of cause-and-effect relationships.
2. Using accurate methods requires a lot of time and resources.
3. There is no way to gather statistical material to use probability theory.
4. There are great difficulties in applying known analytical relationships between the causes (factors of influence) and the corresponding effect, or it is absent at all since these factors are heterogeneous in nature; that is, they can be qualitative (type and condition of the road surface, type of tires) and quantitative (weight of cargo, speed of movement car). In addition, information about quantitative values is often presented in linguistic form.

The evaluation of vehicle traffic parameters is a fundamental component of analyzing emergency situations, and it is primarily grounded in the theory of the car's operational properties^7,8^. From the perspective of traffic safety and the examination of emergency situations, various scientists, including^1,3,5^, have explored the theory of a car's operational properties. The European network of forensic institutions relies on a list of recommended works^9^ for expert practice. Moreover, there is a noteworthy shift towards incorporating modern digital tools and software products into investigative practice. Relevant are the works devoted to the implementation of modern digital tools for the investigation of emergency situations(use of laser scanning of the accident place^1^, laser rangefinders^6^, decelerometers^10,11^, smartphones^11,12^, mobile measuring and registration complexes^13^, other special equipment for performing investigative experiments) and special software products^14^ (photogrammetric programs used to perform all kinds of large-scale measurements of the location of objects at the scene of a road accident, programs for space–time analysis of the movement of vehicles and pedestrians in the conditions of a road accident, graphic editors that allow building large-scale schemes of road accidents, programs for determining the movement parameters of road accident participants in given conditions, a system of visual modeling of the traffic situation) into expert practice. An analysis of innovative developments in the automotive industry^15^ reveals promising avenues for leveraging information from electronic control systems, safety and comfort features in vehicles, to determine the circumstances of road accidents and develop new methods for analyzing emergency situations^6,11,16^. These advancements have been made possible by the development of various technologies, including the Global Positioning System, which allows for vehicle location tracking^17^; event data registration systems integrated into vehicles, enabling the recording of vehicle parameters during accidents^18^; Automatic Crash Notification Systems^19^; Autonomous Driving Systems^20,21^; systems aimed at enhancing the safety of individual vehicles^22–24^; and systems for managing and monitoring traffic flows^25,26^, especially within the context of intelligent transport systems^27^.

The main circumstances of the occurrence of a road accident, according to the current Traffic Rules^28^, are traffic danger and traffic obstruction. In the event of an obstacle to movement, the driver is allowed to apply both braking and maneuvering equally. In the event of a traffic hazard, only brake. From a technical point of view, maneuvering is more difficult and dangerous than emergency braking of a vehicle, and the possibility of combining both processes largely depends on the quality of preparation and professionalism of the driver^29,30^, therefore the main method of preventing road accidents is the braking process^1,4,9^. The effectiveness of the braking process depends on the features of the design and operation of the brake systems of the vehicle (the technical condition of the braking system^31^, the presence of an anti-lock braking system (ABS), and a motion stabilization system (ESP)^32,33^, an emergency braking system Brake Assist (BA)^34^, preventive safety systems^35^, energy recovery systems in hybrid and electric vehicles^36,37^, etc.) and is limited by the number of frictional forces in the contact of the tires with the road^33,38^. Improvements in the design of braking systems and algorithms for their operation have led to a noticeable difference in the braking efficiency of modern vehicles in comparison with the braking efficiency of outdated vehicles, which are in exploitation^1,10,12^. In addition, many developed countries of the world have included the development of electric mobility among the priorities of their transport policy^39^, which has led to the mass distribution of serial production of electric vehicles. These circumstances encourage the revision, updating, and improvement of the existing methodological base for evaluating the braking processes of the entire spectrum of vehicles in exploitation. A fairly large amount of scientific research is dedicated to the study of the processes of ensuring the braking efficiency of electric vehicles. In particular, the researchers focused on such issues as:

- evaluation of emergency braking parameters of electric vehicles using a decelerometer and a smartphone as a new alternative method in order to obtain data that can be useful for forensic practice^11,12^;
- modeling and simulation of automated functions of vehicles in critical situations with the aim of improving existing and forming new methods of reconstruction of traffic accidents^16,40^;
- analysis of the impact of external obstacles and disturbances on the change in energy supply parameters and the functioning of automated vehicle systems^36,37^;
- assessment of the impact of driver reliability on the effectiveness of accident prevention^23,29,30^;
- studying the behavior of automated vehicles in a mixed traffic environment (in the presence of human-operated vehicles) with the aim of reducing accidents and their consequences in the transport network^20^;
- optimization of the trajectory of electric vehicles, taking into account the dynamic properties of the suspension, steering, braking system, and external disturbances during autonomous driving^41,42^.

The purpose of this research is to improve the quality of auto technical examination of road accidents by improving the methodology for evaluating the dynamics of emergency braking of a vehicle and its trajectory.

To achieve the goal, the following tasks were solved:

- to investigate the processes of forming indicators of the dynamics of emergency braking of a vehicle and its trajectory of movement by determining the laws of change of forces and moments that act on it in the DCRE system;
- perform an experimental study of the trajectory of electric vehicles during emergency braking;
- perform a comparative assessment of the stability of the electric vehicle during emergency braking based on the existing and proposed approach in specialized computer programs PC-Crash and Mathcad.

## A mathematical model of the dynamics of emergency braking of a car and its trajectory

Various methods, applications, and technologies^4,7,10,33,35,38^ are used to assess the interaction between tires and the road and the braking properties of vehicles for the purpose of preventing and analyzing road accidents. The selection of these methods depends on the specific research's purpose and direction.

When electronic vehicle systems, which record movement parameters during road accidents, are unavailable, assessing tire-road adhesion qualities can be achieved through investigative experiments^3,9^. Such experiments determine the braking distance, deceleration, or adhesion coefficient, which characterize the traction properties of tires on the road surface. In cases where experiments are not feasible, normative values for braking distance, deceleration, or the coupling coefficient can be used, as outlined in^28,43,44^, or determined based on reference experimental and calculation data^1,9,38^.

During the investigation of traffic accidents, determining the parameters of a vehicle's braking effectiveness involves calculations. Experts rely on measurement results and typical reference data. The expert selects reference data independently, considering the accident conditions. These parameters include driver reaction time, brake system activation delay time, deceleration build-up time, steady deceleration, road surface quality and condition, the coefficient of tire adhesion to the road, and more.

**Figure 1:**
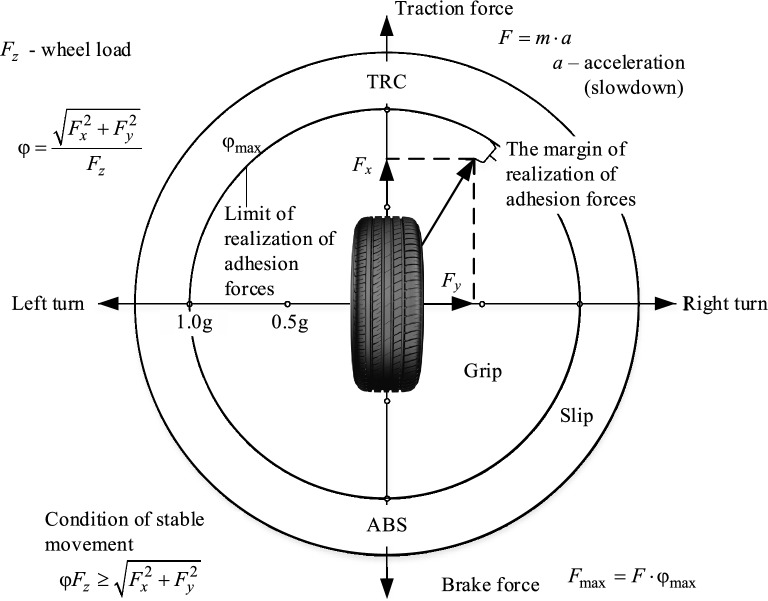
Forces in the tire friction circle.

The mathematical model presented below is an extension of previous works^45,46^ and is developed for the following reasons. A car's stability is influenced by the lateral reactions exerted on its wheels by the road's support surface. Lateral forces can result from centrifugal effects when turning, road cross slopes, wind, etc. If these forces are below the limit value of the lateral reaction, the wheel adheres to the intended trajectory with only minor deviations due to lateral shifts. However, when the lateral reaction exceeds its limit, the wheel loses stability, leading to lateral sliding. Wheel stability (as depicted in Fig. 1) is maintained under specific conditions, as shown in Eq. (1)^7,8,47^

1 $$R_{\Sigma } { = }\sqrt {R_{x}^{2} + R_{y}^{2} } \le \varphi_{\max } \cdot R_{z} ,$$

where $R_{\Sigma }$ is the total normal reaction in the plane of contact with the support surface; $R_{x}$, $R_{y}$, $R_{z}$ are reactions in the longitudinal, transverse, and vertical directions in contact of the support surface with the wheels of the car; $\varphi_{\max }$ is the value of the coefficient of adhesion, which can be realized under adverse conditions.

When emergency braking is employed to prevent an accident, there is a high likelihood of wheel lock-up, resulting in a loss of directional or trajectory stability. The extent of this instability depends on the balance between locked and non-locked wheels.

In cases where the car's longitudinal axis deviates from its straight-line path by 20° or more, the driver loses the ability to rectify the situation using standard vehicle controls^45^. To prevent such scenarios, modern vehicles are equipped with systems like ABS and ESP^1,3,8,11,34^.

**Figure 2:**
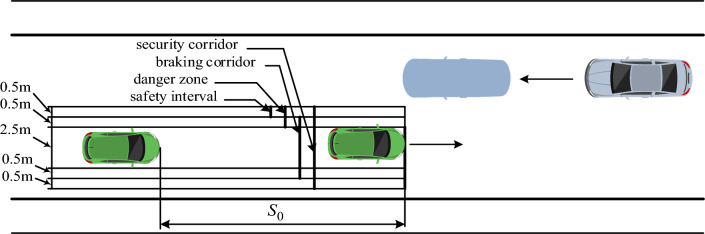
Security corridor.

Regulations outlined in standards^43^ that govern vehicle braking systems allow for variations in the distribution of braking forces among different wheels within certain limits. These standards also account for potential disparities in the operation of braking mechanisms on the wheels of a single axle. While such deviations can result in the vehicle rotating around its center of gravity, the braking process can be deemed safe as long as the dimensions of the braking corridor, with a width of 3.5 m, are maintained (see Fig. 2).

Therefore, the safety of vehicles is ensured by observing the limit values of the braking distance and keeping it within the given traffic lane (safety corridor). The maximum allowable turn of the car is calculated from the condition (see Eq. 2)^45^:

2 $$\frac{{B_{sr} }}{2} \le y + L_{a} \sin \upgamma + \frac{{B_{a} }}{2}\cos \upgamma ,$$

where $B_{sr}$ is the width of the safety corridor (traffic lane); *y* is the deviation of the center of mass of the vehicle in the transverse direction; $L_{a}$ and $B_{a}$ are the length and width of the vehicle; γ is the course angle, which characterizes the position of the longitudinal axis of the vehicle.

The braking process can result in the vehicle moving in a curved path, regardless of the steering wheel's position. This can occur when there is a significant difference in the longitudinal forces acting on the car's wheels due to various operational, technological, or design factors. These variations lead to the generation of turning moments, which can cause the vehicle to turn in the horizontal plane. The influence of these moments can compromise the vehicle's stability, potentially leading to skidding or even overturning.

For a two-axle vehicle, we can distinguish between two stages in the braking process: the dynamic stage and the stage of steady deceleration. The dynamic stage is characterized by an increase in braking forces on the car's wheels, starting from zero and reaching values limited by the wheels' grip on the road surface, which depends on the pressure created in the brake actuator. The dynamic stage during emergency braking typically lasts around 0.5 s, but its duration can vary based on the driver's characteristics, road conditions, and vehicle design. After reaching the maximum deceleration, the stage of established deceleration continues until the vehicle comes to a complete stop. The forces and moments acting on the vehicle during these two braking stages follow significantly different principles^1,3,8,9,44^.

**Figure 3:**
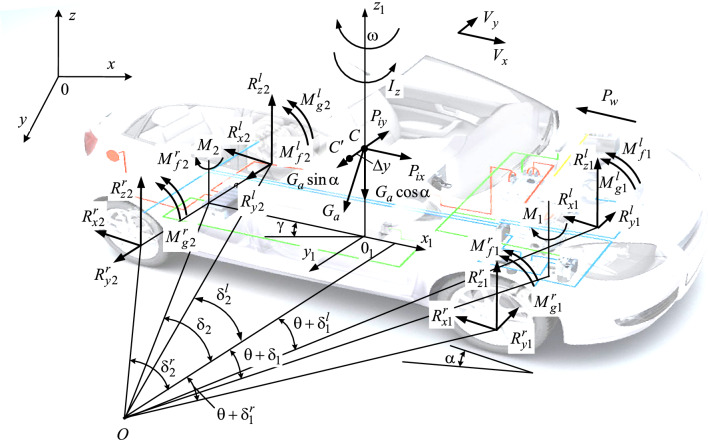
Scheme of forces and moments acting on the vehicle during braking.

The vehicle's position in its surroundings can be determined using coordinates that describe the movement of its center of mass (x, y) within a fixed Cartesian three-dimensional coordinate system (x, y, z), along with the course angle (γ) that indicates the longitudinal axis's rotation (see Fig. 3).

The following notations are used on Fig. 3: $P_{ix}$, $P_{iy}$ are longitudinal and transverse components of the vehicle inertia forces $P_{i}$; $P_{w}$ is the air resistance; $G_{a}$ is the vehicle weight; α is the angle of inclination of the road, $R_{x1}^{l} ,R_{x1}^{r} ,R_{x2}^{l} ,R_{x2}^{r}$ are longitudinal reactions, acting in contact of the wheels with the road surface; $R_{y1}^{l} ,R_{y1}^{r} ,R_{y2}^{l} ,R_{y2}^{r}$ are lateral reactions acting in contact of the wheels with the road surface; $R_{z1}^{l} ,R_{z1}^{r} ,R_{z2}^{l} ,R_{z2}^{r}$ are normal (vertical) reactions acting in contact of the wheels with the road surface; $M_{f1}^{l} ,M_{f1}^{r} ,\;M_{f2}^{l} ,M_{f2}^{r}$ are wheel rolling resistance moments; $M_{g1}^{l} ,M_{g1}^{r} ,\;M_{g2}^{l} ,M_{g2}^{r}$ are braking moments applied to the wheels; Δ*y* is the lateral displacement of the car's center of mass; $M_{1} ,\;M_{2}$ are vehicle turning moments; *I*_*z*_ is the moment of inertia of the vehicle, relative to the vertical axis that passes through its center of mass; ω is the angular speed of the vehicle relative to the axis *z*; $V_{x}$ and $V_{y}$ is the speed of the center of mass, respectively, in the direction of the longitudinal axis of the car and in the direction perpendicular to it; δ_1_ and δ_2_ are angles of shift (side deflection) of the wheels of the front and rear axles; θ is the angle that characterizes the change in the direction of movement of the front axle of the vehicle due to the mismatch of the kinematics of the steering drive and the suspension in the case of a dependent suspension or due to the inclination of the wheels in the case of an independent suspension when the body rolls; indices 1 and 2 indicate front and rear axles, and indices *l* and *r* are left and right wheels.

To determine these parameters, it is necessary to draw up the equilibrium equation (see Eq. 3), which for the braking mode with the clutch disengaged will take the form:

3 $$\left\{ \begin{aligned} \frac{{G_{a} }}{g}\frac{{dV_{x} }}{dt} & = - R_{x1} - R_{x2} - P_{wx} - P_{f} \pm G_{a} \sin \upalpha , \hfill \\ \frac{{G_{a} }}{g}\frac{{dV_{y} }}{dt} & = - R_{y1} + R_{y2} , \hfill \\ I_{z} \frac{d\upomega }{{dt}} & = M_{1} + M_{2} - R_{y1} \cdot a - R_{y2} \cdot b, \hfill \\ \end{aligned} \right.$$

where $\frac{{G_{a} }}{g}\frac{{dV_{x} }}{dt} = P_{ix}$, $\frac{{G_{a} }}{g}\frac{{dV_{y} }}{dt} = P_{iy}$ are projections of the inertia force on the x and y axes; g is the free-fall acceleration; $\frac{{dV_{x} }}{dt}$, $\frac{{dV_{y} }}{dt}$ are acceleration of the vehicle’s center of mass in the direction of the x and y axes; $R_{x1} = R_{x1}^{l} + R_{x1}^{r}$, $R_{x2} = R_{x2}^{l} + R_{x2}^{r}$; $R_{y1} = R_{y1}^{l} + R_{y1}^{r}$, $R_{y2} = R_{y2}^{l} + R_{y2}^{r}$; $M_{1} = \left( {0.5B - \Delta y} \right) \cdot R_{x1}^{r} - \left( {0.5B + \Delta y} \right) \cdot R_{x1}^{l}$; $M_{2} = \left( {0.5B - \Delta y} \right) \cdot R_{x2}^{r} - \left( {0.5B + \Delta y} \right) \cdot R_{x2}^{l}$; *a*, *b*, *B*, *L* are structural parameters of the vehicle; $d\upomega /dt$ is the angular acceleration of the vehicle relative to the axis *z*; $P_{wx} = 0.5 \cdot c_{x} \cdot \uprho \cdot F_{w} \cdot V_{x}^{2}$ is the projection of air resistance on the axis *x*; $c_{x}$ is the coefficient of vehicle streamlining; $\uprho$ is the air density; $F_{w}$ is the cross-sectional area; $P_{f} = f \cdot G_{a} \cdot \cos \upalpha$ is the rolling resistance of the wheels; *f* is the rolling resistance coefficient; $P_{\alpha } = G_{a} \cdot \sin \upalpha$ is the lifting resistance (taken as positive when the car is moving uphill and negative when moving downhill).

Angles of the direction of movement of the wheels ${\updelta }_{1}^{l}$, ${\updelta }_{1}^{r}$, ${\updelta }_{2}^{l}$ and ${\updelta }_{2}^{r}$, are shown in Fig. 3; in the case of an unlocked wheel is the shear angle, and in the case of a locked wheel is the sliding angle. The angle characterizes the change in the direction of movement of the front axle of the vehicle due to the inclination of the wheels when the vehicle body rolls $\theta$. Angle sizes $\theta$ (see Eq. 4) are small and vary from zero to one degree^45^.

4 $$\theta = \rho_{n} \left( { - j_{y} + V_{x} \omega } \right),$$

where $\rho_{n}$ is the parameter that characterizes the structure and elastic properties of the vehicle suspension.

Since the values of the angles $\theta$ during the braking of the vehicle are small (see Eq. 5), it is possible to record

5 $$\theta + \delta_{1}^{r} \approx \theta + \delta_{1}^{l} \approx \theta + \delta_{1} ,\;\;\;\;\delta_{2}^{r} \approx \delta_{2}^{l} \approx \delta_{2} .$$

According to the kinematics of rotation (Fig. 3)^46,47^ obtained equations (see Eq. 6):

6 $${\text{tg}}\,\delta_{{1}} = \frac{{\omega \cdot a - V_{y} }}{{V_{x} }} - \theta ,\;\;\;\;\;{\text{tg}}\,\delta_{{2}} = \frac{{\omega \cdot b + V_{y} }}{{V_{x} }}.$$

The principle of the vehicle movement and the parameters of its trajectory during braking are determined by the laws of change of forces and moments included in the system (see Eq. 3).

When braking the vehicle (see Fig. 3) by the components that form the magnitude of the longitudinal reaction $R_{xk}$ (see Eq. 7) in contact of the wheel with the road surface are the applied braking moment $M_{gk}$, rolling resistance moment $M_{fk}$, moment of inertia of the wheel $M_{jk}$, acting in the opposite direction to $M_{gk}$ and $M_{fk}$, and the dynamic radius of the wheel $r_{d}$

7 $$R_{xk} = \frac{{M_{gk} + M_{fk} - M_{jk} }}{{r_{d} }},$$

where $M_{gk} = P_{gk} \cdot r_{d}$, $P_{gk}$ is the braking force relative to wheel radius; $M_{fk} = P_{fk} \cdot r_{d}$, $P_{fk} = R_{zk} \cdot f$ is the rolling resistance force acting on the wheel, $R_{zk}$ is the normal reaction in contact of the tire with the road; $M_{jk} = J_{k} \cdot \varepsilon_{k}$, $J_{k} = m_{k} \cdot r_{d}^{2}$ is the moment of inertia of the wheel, $m_{k}$ is the wheel weight, $\varepsilon_{k}$ is the angular deceleration of the wheel.

Considering the fact that the implementation of the braking force in contact of the tire with the road is limited by the value of the coefficient of adhesion $P_{gk\max } = R_{zk} \cdot \varphi$, expression (7) will take the form (see Eq. 8)

8 $$R_{xk} = R_{zk} \cdot \left( {\varphi + f} \right) - \frac{{J_{k} \cdot \varepsilon_{k} }}{{r_{d} }}.$$

Having recorded since Fig. 3 equations of equilibrium in the vertical plane, it is possible to determine the sum of the normal reactions acting on the wheels of the car (see Eq. 9), so

9 $$\sum {R_{z} } = G_{a} \cdot \cos \upalpha \pm P_{wz} ,$$

where $P_{wz} = 0.5 \cdot c_{z} \cdot \uprho \cdot F_{z} \cdot V_{x}^{2}$ is the projection of air resistance on the axis *z*; $F_{z}$ is the area of vehicle elements based on which the lifting or downforce is created; $c_{z}$ is the coefficient of lifting or compressive force.

Projection $P_{wz}$ is subtracted from the weight of the vehicle if it creates a lifting force at $c_{z} > 0$, or added to the weight of the vehicle if it creates a downforce at $c_{z} < 0$.

Distribution of the total normal response of vehicle wheels $\sum {R_{z} }$ between its axles, as can be seen from Fig. 3, will depend on the location of the center of mass of the vehicle, the slope of the road, and the forces acting on the vehicle during its movement in braking mode (see Eq. 10)

10 $$R_{z2} = \frac{{G_{a} }}{L} \cdot \left( {a \cdot \cos \alpha + \sin \alpha \cdot h_{g} - \frac{{\delta_{i} }}{g} \cdot j_{x} \cdot h_{g} + \cos \alpha \cdot f \cdot r_{d} } \right),\;\;R_{z1} = \sum {R_{z} } - R_{z2}$$

If there is a displacement of the center of mass of the vehicle Δy, normal reactions on the wheels of the car are distributed as follows (see Eqs. 11 and 12)

11 $$R_{z1}^{l} = R_{z1} \cdot \left( {\frac{1}{2} - \frac{\Delta y }{B}} \right),\;\;R_{z1}^{r} = R_{z1} \cdot \left( {\frac{1}{2} + \frac{\Delta y }{B}} \right),$$

12 $$R_{z2}^{l} = R_{z2} \cdot \left( {\frac{1}{2} - \frac{\Delta y }{B}} \right),\;\;R_{z2}^{r} = R_{z2} \cdot \left( {\frac{1}{2} + \frac{\Delta y }{B}} \right).$$

Therefore, during emergency braking, considering the expression (9), the sum of the longitudinal reactions of the wheels of the vehicle (see Eq. 13) can be determined as

13 $$\sum {R } = \left( {G_{a} \cdot \cos \upalpha \pm P_{wz} } \right) \cdot \left( {\varphi + f} \right) - \sum {\frac{{J_{k} \cdot \varepsilon_{k} }}{{r_{d} }}} .$$

Considering (Eq. 13) and (Eq. 3), the unfolded equation of motion of the vehicle during braking (see Eq. 14) will have the form

14 $$\frac{{G_{a} }}{g}j_{x} \cdot \updelta_{j} = - \left( {G_{a} \cdot \cos \upalpha \pm P_{wz} } \right) \cdot \left( {\varphi + f} \right) - P_{wx} \pm G_{a} \sin \upalpha ,$$

where $\updelta_{j}$ is the coefficient that considers the inertia of the rotating masses of the vehicle^43^.

Let's express the acceleration of the vehicle during emergency braking from Eq. (14) and write the resulting expression (see Eq. 15) as a differential equation to ensure the possibility of considering the change of the speed of the vehicle during braking in time

15 $$\frac{{dV_{x} }}{dt} = - \frac{g}{{G_{a} \cdot \updelta_{j} }}\left[ {\left( {G_{a} \cdot \cos \upalpha \pm P_{wz} } \right) \cdot \left( {\varphi + f} \right) - P_{wx} \pm G_{a} \sin \upalpha } \right].$$

After substituting the force values $P_{wx}$, $P_{wz}$ and some transformations, Eq. (16) can be obtained

16 $$\frac{{dV_{x} }}{dt} = - \frac{g}{{\updelta_{j} }}\left[ {\left( {\cos \upalpha \pm \frac{{0.5c_{z} \uprho F_{z} V_{x}^{2} }}{{G_{a} }}} \right) \cdot \left( {\varphi + f} \right) - \frac{{0.5c_{x} \uprho F_{w} V_{x}^{2} }}{{G_{a} }} \pm \sin \upalpha } \right].$$

Lateral reactions acting in contact of the wheels with the road surface $R_{y1}^{l}$, $R_{y1}^{r}$, $R_{y2}^{l}$, $R_{y2}^{r}$, can be determined from a known dependence (see Eq. 17)^47^

17 $$R_{y} = k_{y} {\text{tg}}\updelta ,$$

where ${\updelta }$ is the angle of shift (side deflection) of the wheel; $k_{y}$ is the coefficient of resistance to the lateral shift (retraction) of the wheel.

The law of change $k_{y}$ can be approximated using empirical formulas proposed by R. Smiley and V. Horn^48^, verified in^45^. For automobile tires (except for tires with adjustable air pressure), according to the results of calculations using these formulas, a reasonably good approximation of calculated and experimental data is achieved (see Eq. 18). Thus, $k_{y}$ can be defined as

18 $$k_{y} = C_{c} \left( {A - \frac{E}{{p_{w} }}} \right),$$

where $C_{c} = 1.038 \cdot 10^{ - 4} \cdot G_{k}^{2} - 0.168 \cdot G_{k} + 141.062$; $G_{k}$ is a wheel load; $A = 0.714G_{k} \sqrt {\frac{{B_{t}^{2} }}{{D_{0}^{3} }}} ;$ $E = 2.2\frac{{G_{k}^{2} B_{t} }}{{D_{0}^{3} }};$ $B_{t}$ is the width of the profile of the undeformed tire; $D_{0}$ is a free diameter of the tire.

Equation (18) is valid for $p_{w} \ge 4.77\frac{{G_{k} }}{{\sqrt {D_{{_{0} }}^{3} B_{t} } }}.$ When $p_{w} \le 4.77\frac{{G_{k} }}{{\sqrt {D_{{_{0} }}^{3} B_{t} } }}$ it is necessary to use the formula (see Eq. 19)

19 $$k_{y} = C_{c} \left( {A^{\prime}p_{w} - E^{\prime}} \right),$$

where $$A^{\prime} = 0.095B_{t}^{2} ;$$ $$E^{\prime} = 0.206\frac{{G_{k} }}{{\sqrt {_{t} D_{0}^{3} } }}.$$

For a locked wheel (see Eq. 20), the value of lateral reactions is

20 $$R_{y1} = R_{x1} \cdot tg\updelta_{1} = R_{z1} \cdot \varphi_{y} \cdot tg\updelta_{1} ,\;\;\;R_{y2} = R_{x2} \cdot tg\updelta_{2} = R_{z2} \cdot \varphi_{y} \cdot tg\updelta_{2} .$$

The parameters characterizing the trajectory of the vehicle during braking can be determined from the system of equilibrium equations (3) based on the established laws of change of forces and moments (see Eq. 21), transforming it into the form

21 $$\left\{ \begin{gathered} \frac{{dV_{x} }}{dt} = f_{{V_{x} }} \left( {V_{x} ,\;\;V_{y} ,\;\;\upomega } \right), \hfill \\ \frac{{dV_{y} }}{dt} = f_{{V_{y} }} \left( {V_{x} ,\;\;V_{y} ,\;\;\upomega } \right), \hfill \\ \frac{d\upomega }{{dt}} = f_{\upomega } \left( {V_{x} ,\;\;V_{y} ,\;\;\upomega } \right). \hfill \\ \end{gathered} \right.$$

System (21) can be solved by numerical methods (for example, Euler's method). This will make it possible, after integration, to obtain functional dependencies of changes in vehicle speeds $V_{x} ,\;\;V_{y} ,\;\;\upomega$ during braking over time.

Re-integration of the obtained dependencies for $V_{x} ,\;\;V_{y} ,\;\;\upomega$ in order to obtain the trajectory of the movement of the center of mass of the vehicle (*x*, *y*) and the reversal of its longitudinal axis γ (see Eq. 22) must be carried out in accordance with the expressions

22 $$x = \int\limits_{0}^{t} {\left( {V_{x} \cos \upgamma + V_{y} \sin \upgamma } \right)\,dt,} \;\;y = \int\limits_{0}^{t} {\left( {V_{x} \sin \upgamma - V_{y} \cos \upgamma } \right)\,dt} ,\;\;\upgamma = \int\limits_{0}^{t} {\upomega \,dt} .$$

The verification of the developed theoretical propositions was carried out based on field experiments (item 3) and a comparison of the results of modeling in the Mathcad environment with the solution of the same problem in the PC-Crash program (item 4).

## Experimental evaluations of the trajectory of electric vehicles during emergency braking

The purpose of this experimental research is to validate the theoretical findings outlined in "A mathematical model of the dynamics of emergency braking of a car and its trajectory" section by comparing simulation results with experimental data and calculations based on the current methodology recommended by the European Network of Forensic Institutions^9^.

Indicators to be fixed: initial speed of vehicle braking, vehicle deceleration, initial direction of movement, braking time, movement in the longitudinal and transverse planes, and course angle. Measuring equipment: measuring and registration complex for testing mobile machines and its elements VDVMM 4-001^13^, laser rangefinder SNDWAY SW-T100.

**Figure 4:**
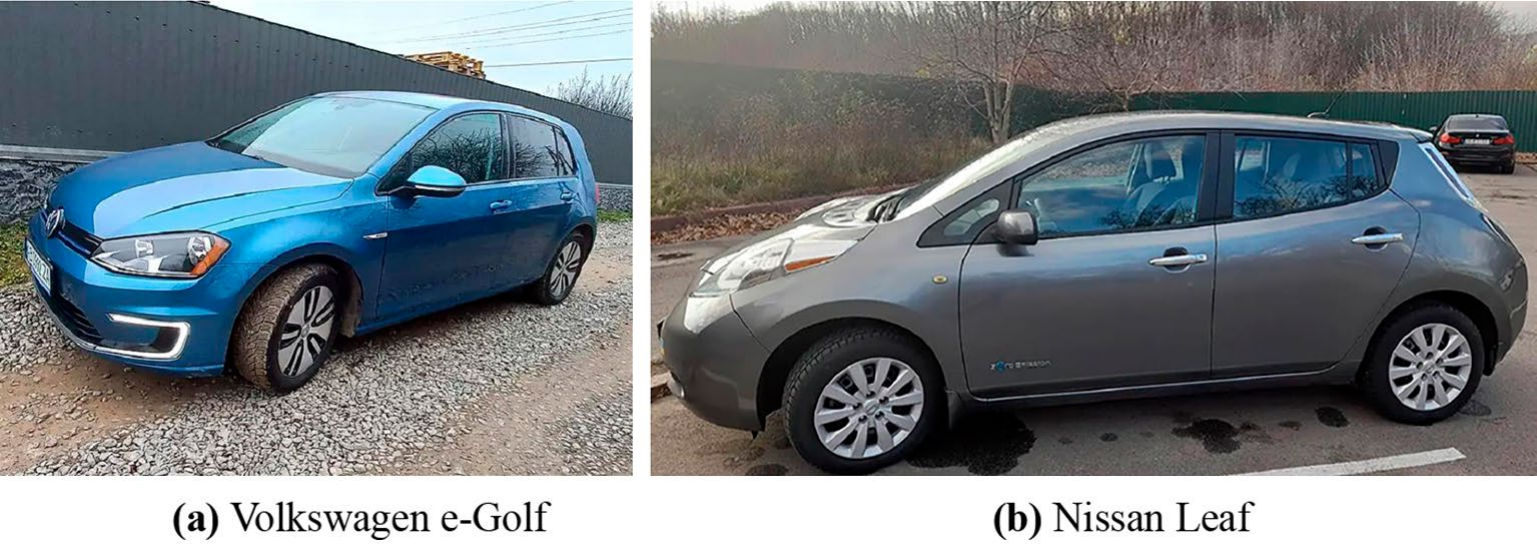
The general appearance of the electric vehicles that participated in the tests.

**Figure 5:**
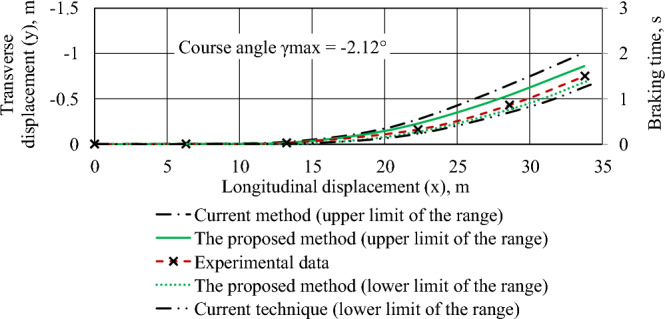
The trajectory of the center of mass of a braked Volkswagen e-Golf car with uneven braking moments.

**Figure 6:**
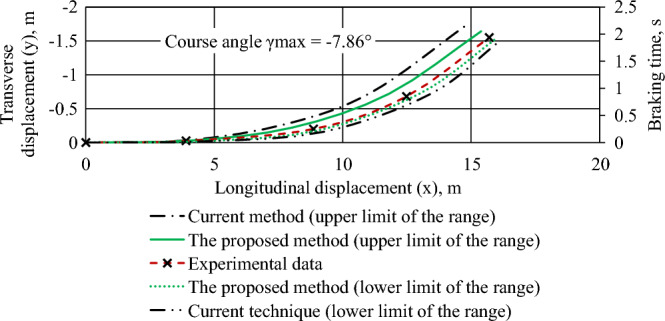
The trajectory of the center of mass of a braked Nissan Leaf car with an uneven distribution of adhesion coefficients on the sides.

**Figure 7:**
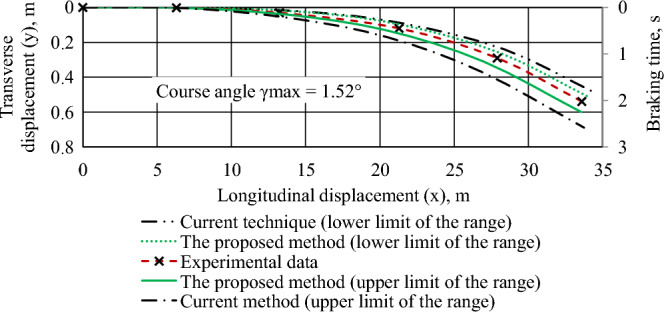
The trajectory of the center of mass of the braked Volkswagen e-Golf car during its lateral displacement.

The experiment was conducted using electric vehicles, specifically the Volkswagen e-Golf and Nissan Leaf, as depicted in Fig. 4. The tests were carried out on a section of asphalt concrete pavement with a horizontal profile, where surface conditions included dry, wet, and dirt-covered surfaces. We studied the effects of uneven braking force distribution, the coefficient of adhesion between the car's wheels, and the transverse displacement of the car's center of mass, with the ABS system disabled. The results of the experimental tests were processed according to the methods outlined in^13^, and you can view examples of the obtained experimental curves of the braked vehicle's movement, as well as the results of modeling based on both the proposed and current methodologies, in Figs. 5, 6, 7.

The initial data for Fig. 5: initial braking speed of 50 km/h; adhesion coefficient—0.7 for all wheels; braking torques on the wheels of the car, N m: front left—465, front right—395, rear—368; negative values of heading angle and lateral displacement indicates the displacement of the vehicle to the left.

For Fig. 6: initial braking speed of 30 km/h; clutch ratio: left wheels—0.7; right wheels—0.38 (mud-covered asphalt concrete); braking torques on the wheels of the car, N m: front—945, rear—828; negative values of heading angle and lateral displacement indicates the displacement of the vehicle to the left.

To ensure the lateral displacement of the vehicle's center of mass, a load weighing 200 kg was evenly placed on its left side at 0.3 m from the longitudinal axis of the car. The initial braking speed is 50 km/h, the coupling coefficient is 0.7 (for all wheels); braking torques on the wheels of the vehicle, N·m: front—794, rear—748.

Comparison of simulation results (according to the proposed method) and experimental data showed that the average relative error is 4.58%, and the maximum error did not exceed 7.82%.

## Research of stability of electric vehicle movement during emergency braking in specialized computer programs PC-Crash and Mathcad

Let's address the problem of studying the stability of the Volkswagen e-Golf during emergency braking using the mathematical models presented above. The initial data for this scenario include the car's mass (*m* = 1585 kg), moment of inertia (*I* = 1829 kg m^2^), movement on a horizontal surface with a coefficient of wheel-road adhesion (φ = 0.8, dry asphalt), the initial car speed ($V_{0}$ = 40 km/h), and angular velocity ($\upomega_{0}$ = 2.5 s^−1^). Such motion could be a result of a collision (post-separation phase of vehicles) or due to skidding on the road, uneven braking moments, variations in adhesion coefficients on each side of the vehicle (e.g., the right side moving toward the curb during braking), and more.

**Figure 8:**
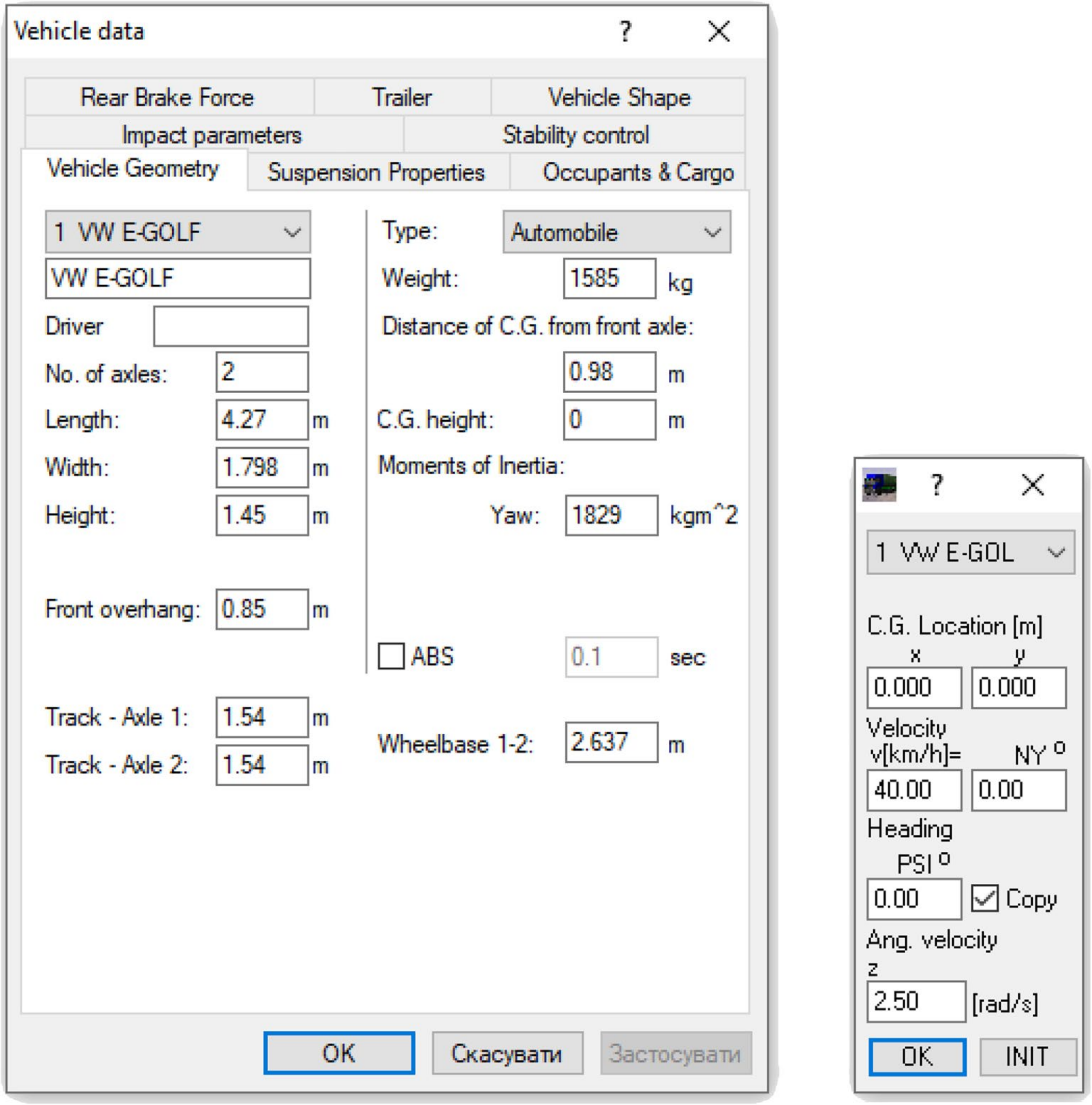
Initial data for modeling.

**Figure 9:**
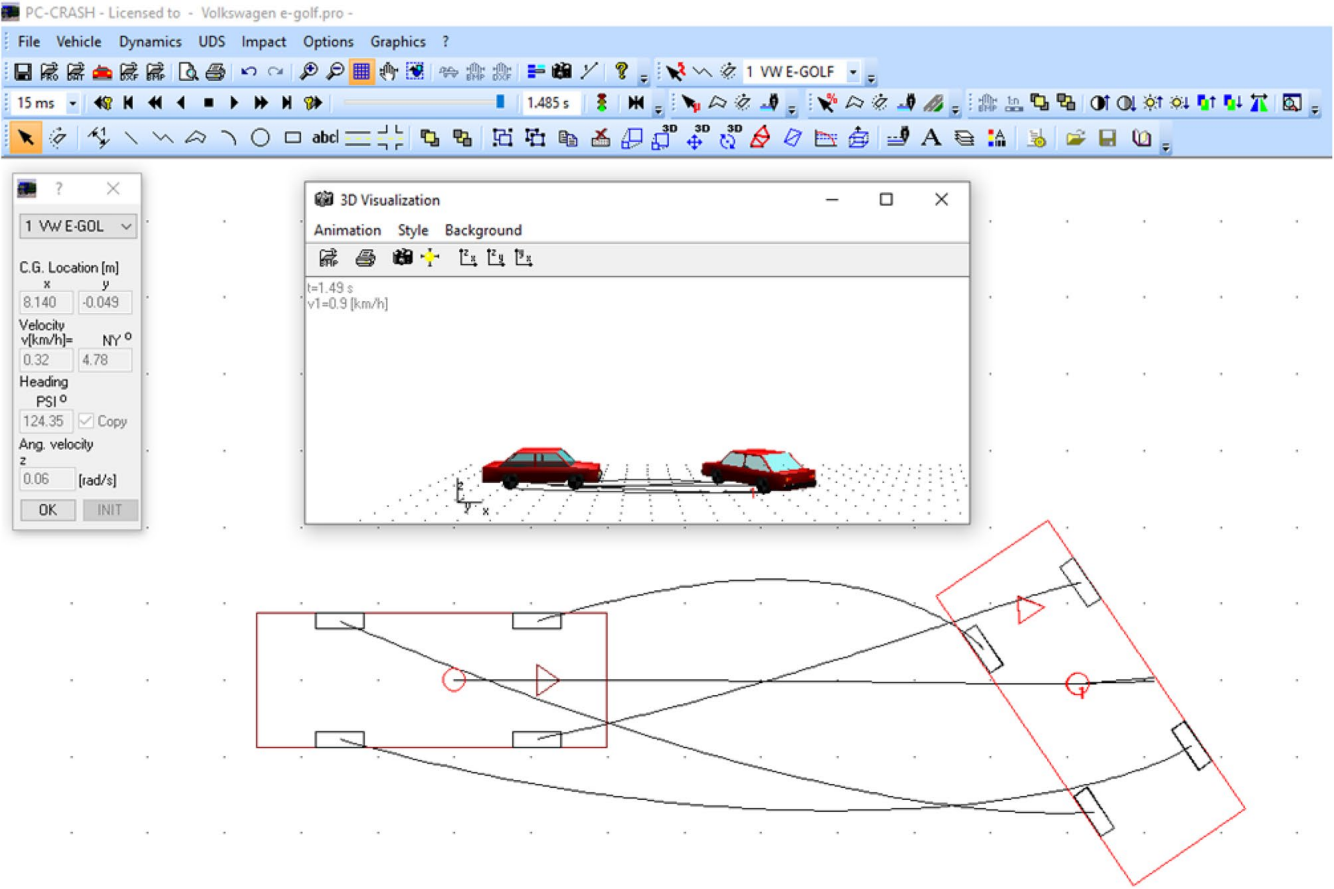
The results of the study of the stability of the Volkswagen e-Golf car during emergency braking.

Figures 8 and 9 display the input data and the results of solving this problem using the PC-Crash program^49^. PC-Crash is recommended for use by the European network of forensic institutions^9^.

In Fig. 8, the left side of the table contains the vehicle's parameters, while the initial speeds are shown on the right. In Fig. 9, the points represent a coordinate grid with a 1-m step. At the initial moment, the vehicle's center of gravity is at coordinates x = 0 and y = 0. The angle (ψ) between the vehicle's longitudinal axis and the x-coordinate axis (oriented to the right in Fig. 8) ψ is 0°.

**Figure 10:**
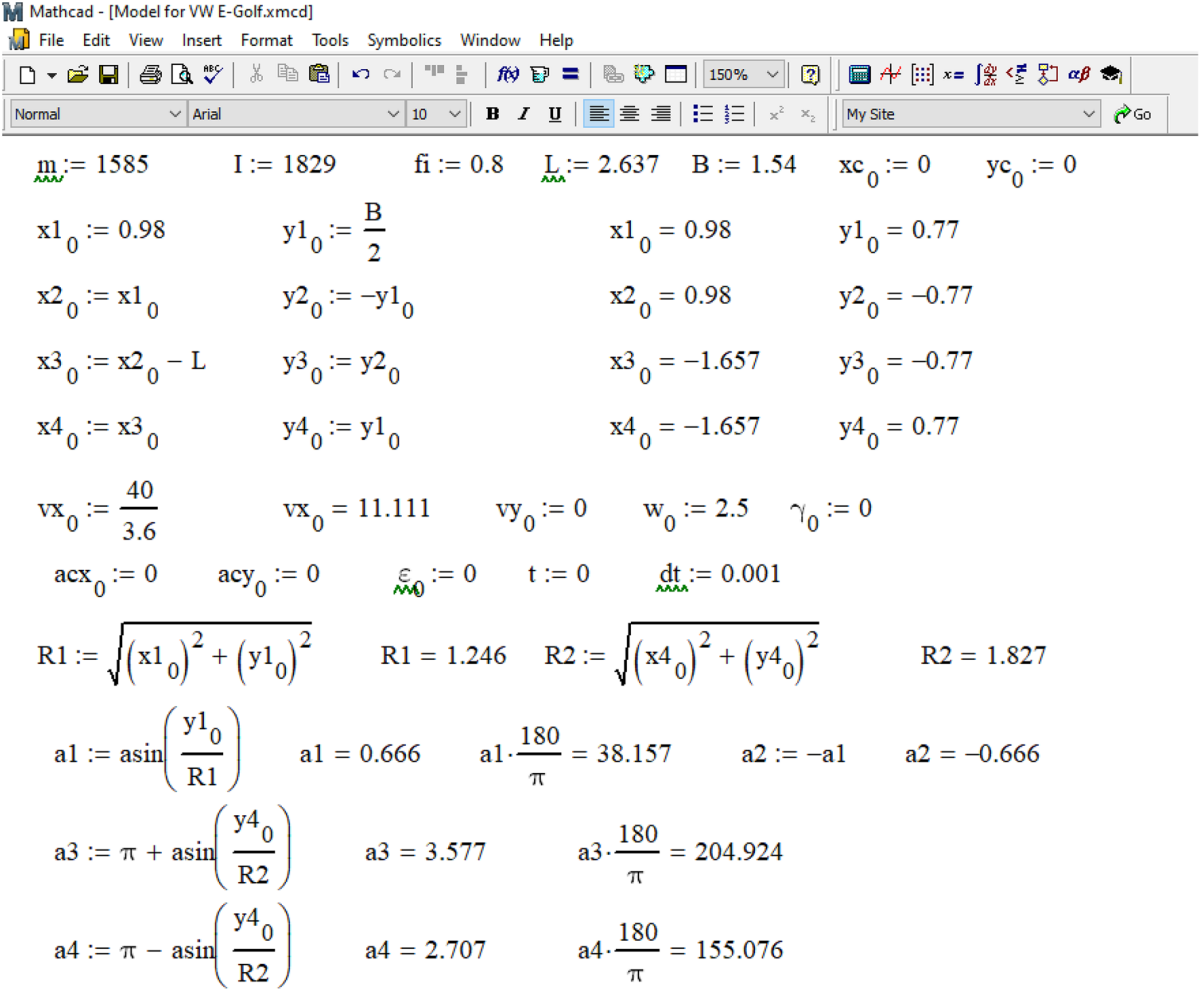
Formation of output data in the Mathcad environment for the simulation of Volkswagen e-Golf movement during emergency braking.

Now, let's move on to consider the possibility of solving the problem using the mathematical models presented above in the Mathcad environment. Figure 10 shows the result of generating output data for the simulation of the Volkswagen e-Golf movement during emergency braking.

As can be seen from Fig. 10, the initial value of time t = 0 is set, and the time integration step dt = 0.001 s. The values of the accelerations of the vehicle's center of gravity along the x and y coordinate axes and the angular acceleration around the z-axis at the initial moment of time are unknown, so their values are assumed to be zero:$acy_{0} = 0$, $\varepsilon_{0} = 0$. The wheels of the vehicle are marked as follows: 1 is the front left, 2 is the front right, 3 is the rear right, 4 is the rear left. The initial coordinates of its wheels are determined based on the data in the vehicle parameters table (see Fig. 8).: $x1_{0} = 0.98$ m, $y1_{0} = 0.77$ m, $x2_{0} = 0.98$ m, $y2_{0} = - 0.77$ m, $x3_{0} = - 1.657$ m, $y3_{0} = - 0.77$ m, $x4_{0} = - 1.657$ m, $y4_{0} = 0.77$ m. The lengths and angles with the x-axis of the radius vectors of all wheels are also set. For front wheels R1 = 1.246 m, and for rear wheels R2 = 1.827 m. The angles of the radius vectors of all wheels with the axis *x*: a1 = 0.666 rad (38.157°), a2 = –0.666 rad (–38.157°), a3 = 3.577 rad (204.924°), a4 = 2.707 rad (155.076°).

The motion of the vehicle in the plane is determined by Newton's second law in the form of differential equations of the second order (3). The first two equations in the system (3) describe the movement of the car's center of gravity relative to the x and y axes, and the third describes the car's rotation around its center of gravity (z-axis).

**Figure 11:**
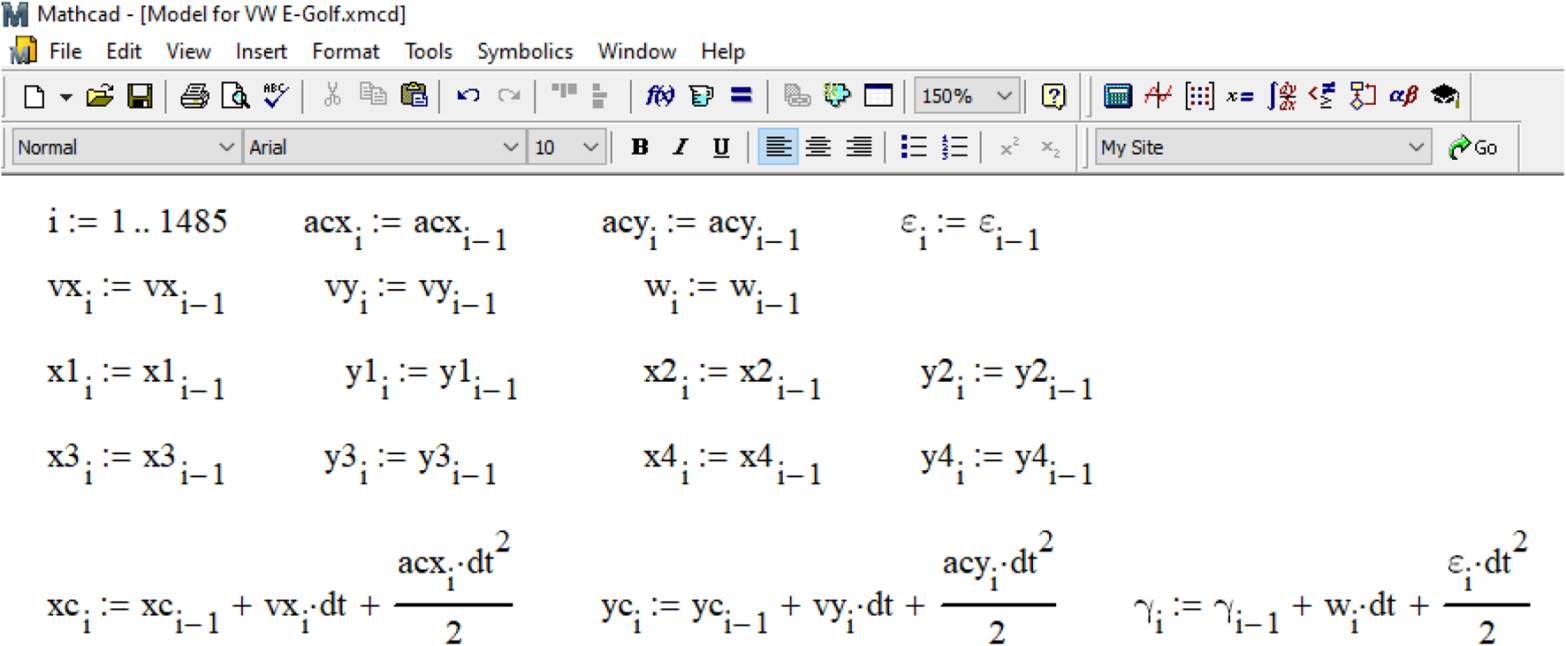
Organization of the calculation cycle.

The calculation cycle for determining the coordinates of the center of gravity of the car and its course angle based on the differential movement equations (3) is organized according to the data in Fig. 11. The time step in this cycle is 0.001 s in the interval from 0 to 1.485 s (like the calculation process in the PC-Crash, see Fig. 9).

Within the cycle, at the i-th step, the values of accelerations and speeds of the vehicle and the coordinates of its wheels are obtained from the previous (i-1) step. These values are then used to calculate the position of the center of gravity and the current course angle at the current step through analytical integration of the differential movement equations. The goal is to determine the direction of movement of the vehicle's wheels and the direction of application of traction force vectors to them. Any delay in determining the direction of the coupling forces caused by the integration step size is compensated by the small size of the step.

**Figure 12:**
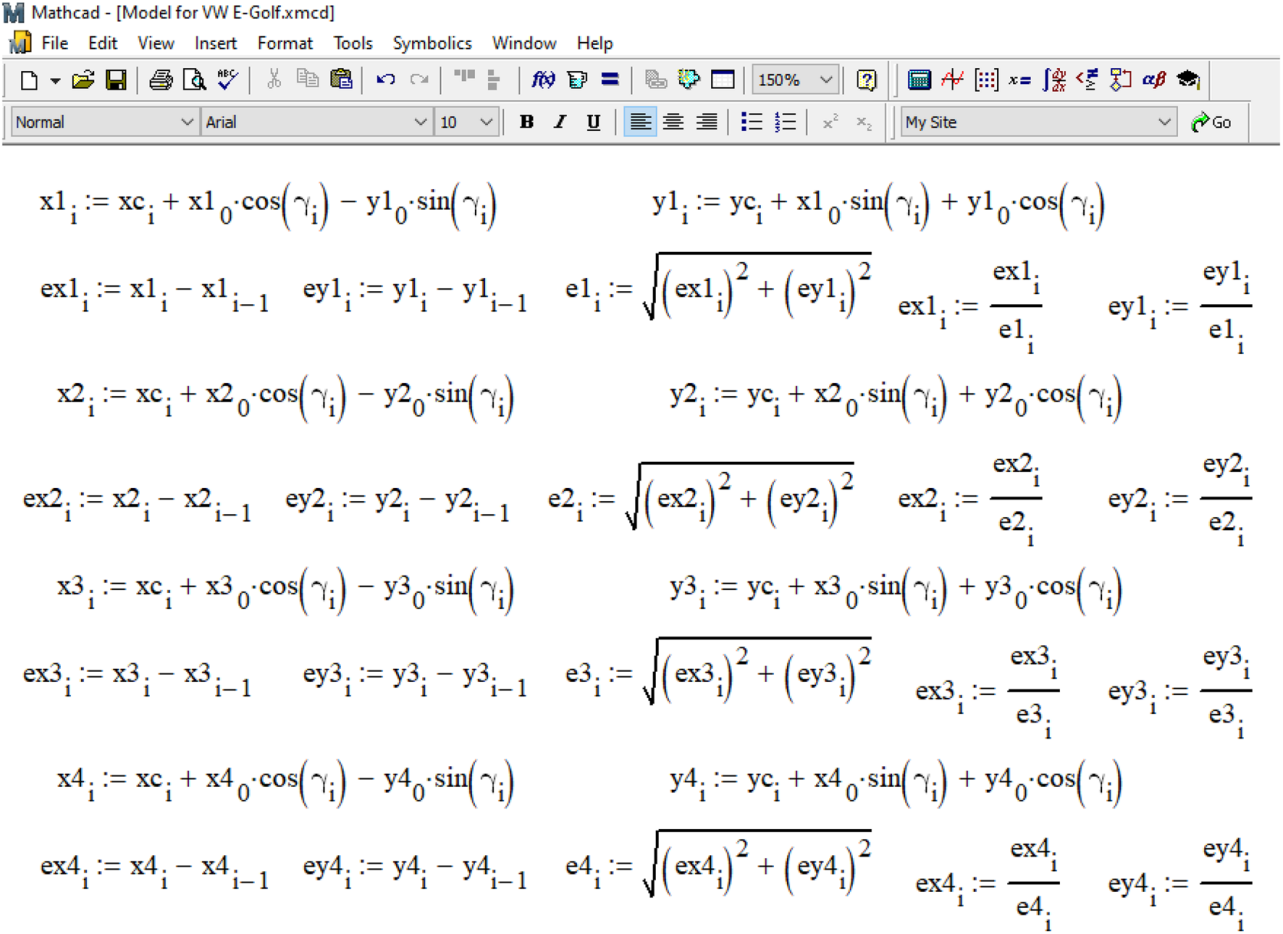
Determination of the current coordinates of each wheel.

As illustrated in Fig. 12, based on the current position of the vehicle's center of gravity and its course angle, the coordinates of each car wheel and the unit vectors (often referred to as 'orts' in analytical geometry) indicating the direction of movement of each wheel are determined. Clutch forces are applied in the opposite direction to the movement of each wheel. The magnitude of the force of adhesion of the car's wheels to the road is determined based on the center of gravity's location, assuming an even distribution of the coefficient of adhesion between the tires and the road across all the vehicle's wheels.

The magnitude of the force of adhesion of the wheels of the car to the road was determined considering the location of its center of gravity and the assumption that the coefficient of adhesion of the tires to the road is distributed evenly between the wheels of the vehicle.

In practical calculations, experts can choose different ratios, such as determining the coupling coefficient for each wheel separately using a neuro-fuzzy model^38^. The projections of the traction forces on each wheel along the axes of the global coordinate system are determined by multiplying the specified force by the components of the wheel's movement direction with a 'minus' sign.

**Figure 13:**
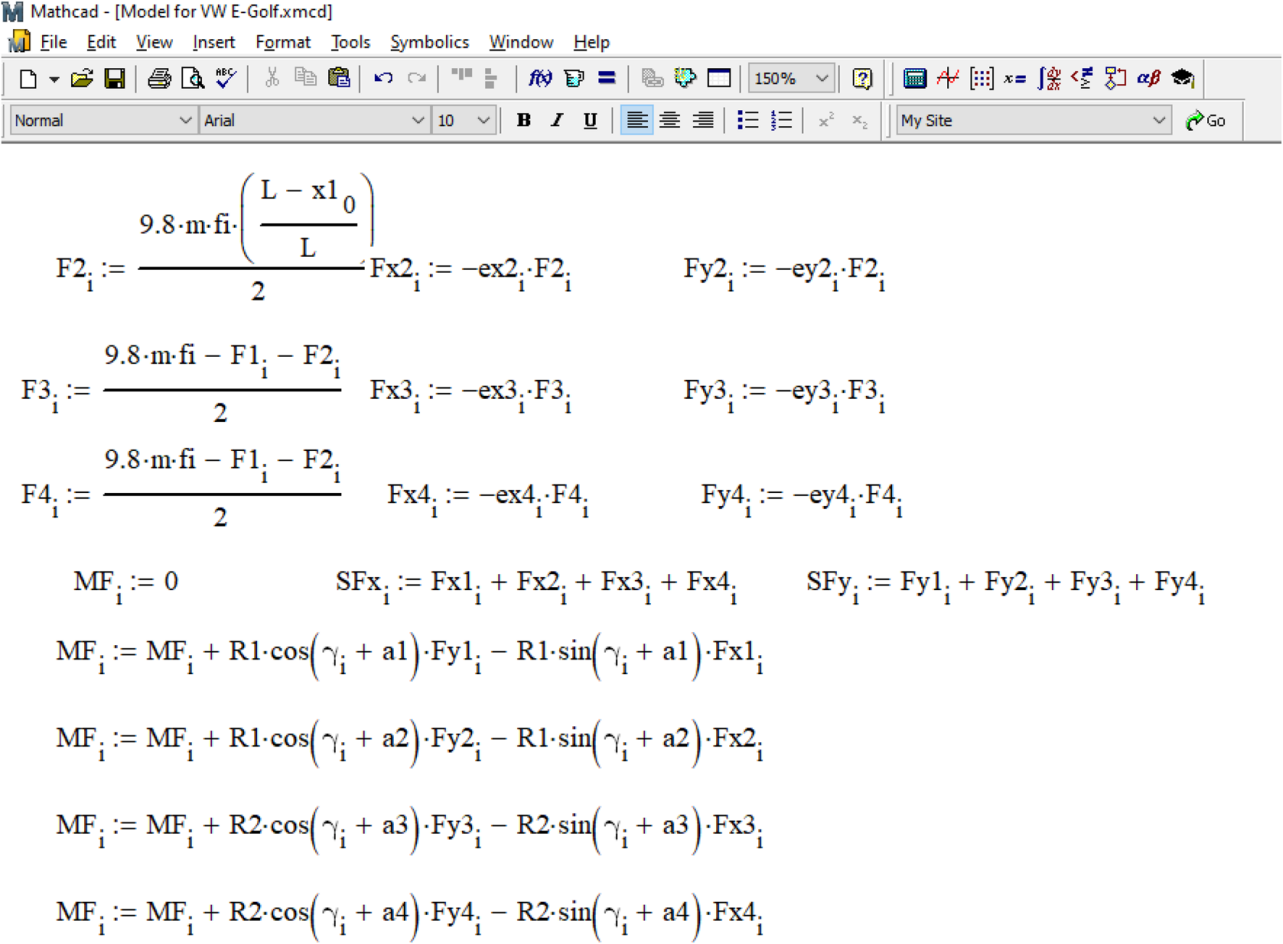
Determination of the moment of the forces acting on the vehicle relative to its center of gravity.

Subsequently, as shown in Fig. 13, the projections of all forces along the coordinate axes are summed. The moments of these forces relative to the center of gravity are calculated as the vector product of the coupling forces and the radius vector of each wheel.

**Figure 14:**
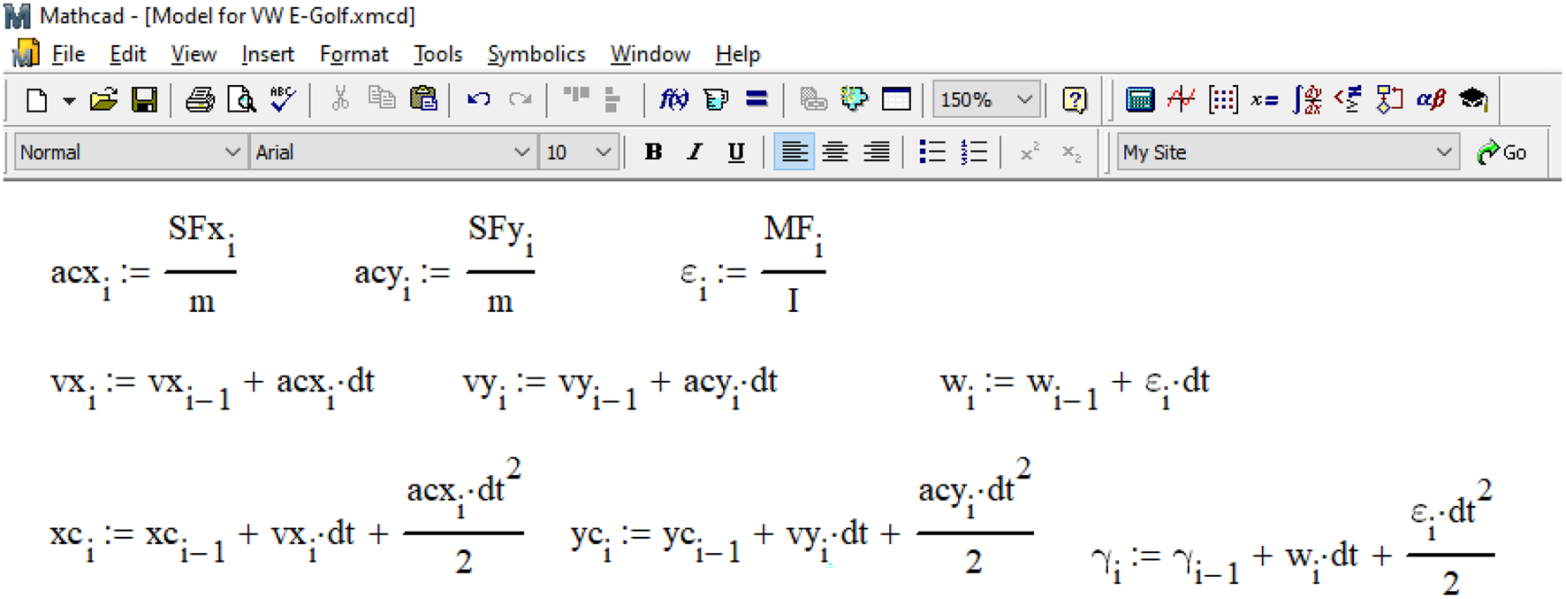
Determination of accelerations, speeds, coordinates of the center of gravity of the vehicle and its course angle.

The current acceleration (or deceleration) of the car's center of gravity and its angular acceleration (or deceleration) are determined based on Newton's second law. Using these data, we calculate the real velocities of the center of gravity in projection on the coordinate axes and the car's rotational speed. From these values, we determine the real coordinates of the center of gravity and the car's course angle at the current step. We consider these values as 'real' because they are based on information carried over from the previous integration step. You can find the procedure for determining these values in Fig. 14.

**Figure 15:**
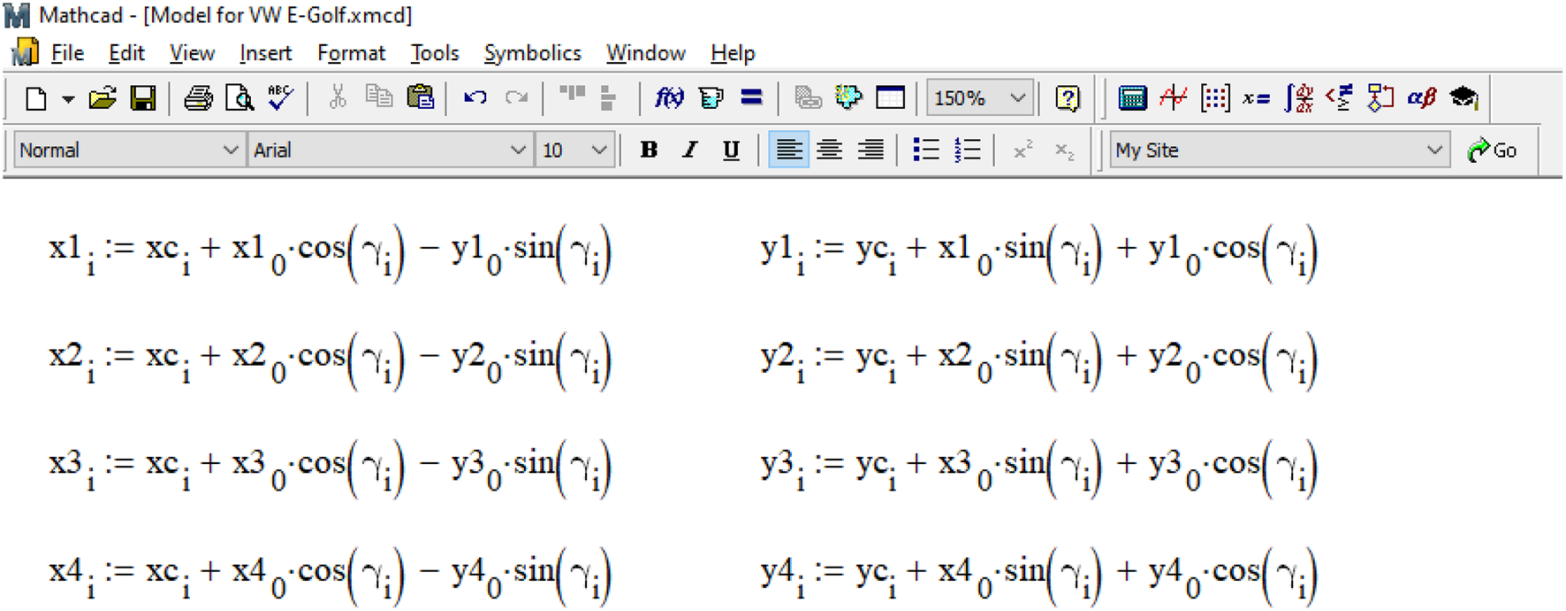
Determining the actual position of the vehicle wheels at the current integration step.

To determine the position of the wheels at the current step, we sequentially calculated the expressions shown in Fig. 15, thereby completing the integration cycle.

**Figure 16:**
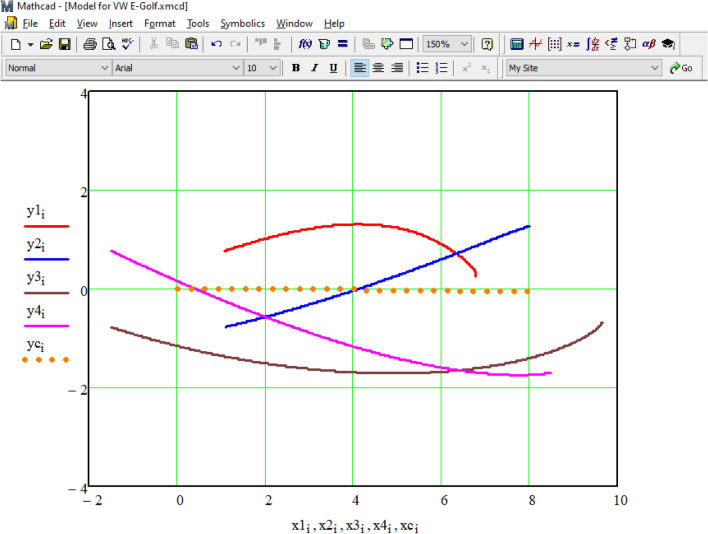
The trajectories of the wheels of the vehicle according to the simulation results.

As a result of the integration performed in the Mathcad environment, we obtain a data table consisting of 1485 rows, with the number of columns corresponding to the calculated values labeled with the subscript 'i.' The visualization of this table, showing the calculated positions of the vehicle's wheels and its center of mass at various moments in time, is presented in Fig. 16. In the figure, the trajectory of the center of mass is represented by dashed orange lines, while the center of mass of the left front wheel is shown in red, the right front wheel in blue, the center of mass of the right rear wheel in purple, and the left rear wheel in brown.

**Figure 17:**
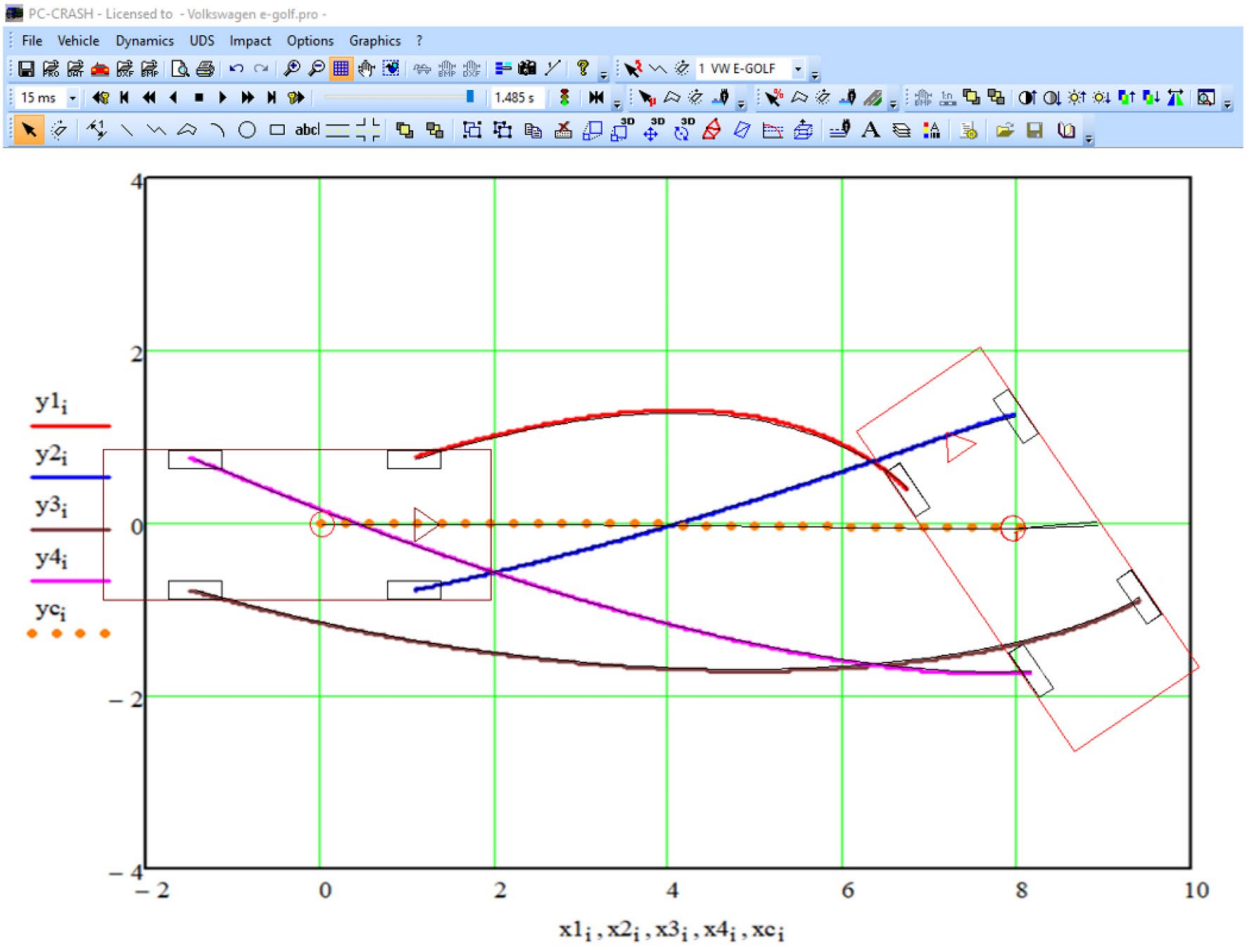
Comparison of simulation results in the Mathcad environment with the solution of the same task in the program PC-Crash.

To compare the simulation results obtained with PC-Crash for the same task, we conducted a comprehensive comparison of the wheel trajectories of the vehicle using the PC-Crash program (as shown in Figs. 9 and 16). The results of this comparison are presented in Fig. 17.

A comparison of the results shows that the wheel trajectories obtained by different methods completely coincide.

## Discussion of the research results

It is important to note that the developed mathematical model is suitable for analyzing the dynamics of emergency braking and the formation of the movement trajectory only in two-axle vehicles without disconnected or inoperable electronic systems for improving traffic safety (ABS, ESP, BA, etc.). Otherwise, the model requires refinement and adaptation to the structural and functional features of the studied vehicles.

The results of the experimental assessment of the emergency braking dynamics of the Volkswagen e-Golf and Nissan Leaf (Figs. 5, 6, 7, "Experimental evaluations of the trajectory of electric vehicles during emergency braking" section) correlate well with the simulation results. The average relative error is 4.58%, and the maximum error does not exceed 7.82%. These results align with findings from researchers at the Kharkiv National Automobile and Road University (Ukraine), Žilina University (Slovak Republic), West-Saxon University Zwickau (Germany), and Zagreb University (Croatia), who are engaged in research related to road accident examination^1,11,12^.

Modeling the trajectory of the Volkswagen e-Golf under braking in the Mathcad environment and comparing the obtained results with the PC-Crash program confirmed the suitability of the methodology recommended for use in the European network of forensic institutions^9^ and the proposed methodology for the analysis of emergency situations with electric vehicles. The developed mathematical model, in contrast to the existing ones^1,3,9,40,46,50–52^, makes it possible to investigate the stability of the vehicle movement during emergency braking as part of the analysis of real emergency situations, when it is necessary to take into account certain circumstances and features, which cannot be taken into account when modeling in specialized software. For example, when the position of the wheels of the vehicle after the impact is significantly different from their position before the impact, in the case of separation of one of the wheels, when one or more wheels were lowered or jammed by deformed parts of the vehicle, during the analysis of the movement of a braked vehicle with an uneven distribution of clutch forces^53,54^, etc.). The analysis of incorporating such features into the modeling process reveals a reduction in the probability of type I errors by 2–19% and type II errors by 43–68%. This reduction is crucial for arriving at objective conclusions in the field of auto-technical examination of traffic accidents.

## Conclusions

This article presents the research results concerning the effectiveness of electric vehicle braking in determining accident circumstances. To assess the dynamics of emergency braking of a vehicle and its trajectory, it is necessary to determine the principles governing the changes in forces and moments acting on the vehicle during the braking process. The main factors that significantly affect the change in the trajectory of the car during braking are the uneven action of the braking moments, the transverse displacement of the center of mass of the car, and the uneven realization of the potential of the car's wheels with the road surface. The developed mathematical model allows for the consideration of various types of uncertainty when setting braking parameters, reducing the range of possible modeling errors by 39%.

Results from the experimental evaluation of electric vehicle trajectories during emergency braking confirm the feasibility of modeling based on the developed models and algorithms. A comparison of the simulation results using the proposed method and experimental data showed that the average relative error is 4.58%, and the maximum error did not exceed 7.82%.

The performed study of the stability of the electric vehicle movement during emergency braking using developed mathematical models in the Mathcad software environment reveals the content of the algorithm of a similar calculation in specialized computer programs for road accident examination (in particular, PC-Crash) and can be used in the absence of specialized software at the disposal of the expert. Performing this kind of calculation is relevant during the analysis of real accident situations when it is necessary to consider certain circumstances and features that cannot be taken into account during modeling in specialized software; at the same time, the probability of occurrence of type I errors is reduced by 2–19%, and type II errors by 43–68%.

Further possible directions of research on this issue may be:

- taking into account the violation of the geometry of the axes in the developed model;
- considering the delay time of the brake actuator activation and the deceleration growth time for each wheel during braking;
- adapting the developed model to evaluate the trajectory of movement during braking for multi-axle trucks;
- improving modeling algorithms to account for the operation of electronic systems for enhancing traffic safety and energy recovery and redistribution systems in electric vehicles.

## Data Availability

The data that support the findings of this study are available from the corresponding author upon reasonable request.
